# Supplementary material for: Lineage–specific amino acids define functional attributes of the protomer-protomer interfaces for the Rad51 and Dmc1 recombinases
Source: bioRxiv. 2024 Dec 4:2024.12.03.626531. Preprint. [Version 1] doi: 10.1101/2024.12.03.626531 (PMC11642858; doi:10.1101/2024.12.03.626531)
Supplement: Supplement 1 [file NIHPP2024.12.03.626531v1-supplement-1.pdf]

bioRxiv preprint doi: https://doi.org/10.1101/2024.12.03.626531; this version posted December 4, 2024. The copyright holder for this preprint (which was not certified by peer review) is the author/funder, who has granted bioRxiv a license to display the preprint in perpetuity. It is made available under aCC-BY 4.0 International license.

Figure S1. Cryo-EM data processing for the Rad51 and Dmc1 nucleoprotein filaments. **a,** Representative micrograph used for Rad51 filament data processing. **b,** Representative 2D classes. **c,** Final 3D map for the Rad51 filament. **d,** Fourier shell correlation curve for the Rad51 data. **e,** Representative micrograph used for Dmc1 filament data processing. **f,** Representative 2D classes. **g,** Final 3D map for the Dmc1 filament. **h,** Fourier shell correlation curve for the Dmc1 data.

Figure S2. Genetic & biochemical analysis of YY to EK mutants. a, Spot assays using 10-fold serial dilutions of *S. cerevisiae* strains expressing *rad51* Y112E, Y253K, Y112E + Y253K or Y112S + Y253L, as indicated, and grown on YPD plus 0.03, 0.015, or 0.005% MMS. In each case, the indicated *rad51* mutant was integrated into chromosome V as depicted in Fig. 4a. **b,** Spot assays using the indicted *rad51* mutant strains plus a plasmid containing the same *rad51* gene to allow for protein overexpression (O/E) and grown on

bioRxiv preprint doi: https://doi.org/10.1101/2024.12.03.626531; this version posted December 4, 2024. The copyright holder for this preprint (which was not certified by peer review) is the author/funder, who has granted bioRxiv a license to display the preprint in perpetuity. It is made available under aCC-BY 4.0 International license.

synthetic dropout media minus Tryptophan (SD -Trp) plus 0.03, 0.015, or 0.005% MMS, as indicated. **c,** D-loop assays with the indicated Rad51 proteins. **d,** Graphical representation of normalized D-loop formation. Data were all normalized to reactions with wild-type Rad51 and bars for the mutant proteins correspond to the mean and stand deviation of three separate experiments. **e,** Single molecule Rad51 filament assembly assays on ssDNA bound by GFP-RPA; the loss of GFP-RPA signal intensity is a proxy for Rad51 filament assembly. **f,** Quantitation of Rad51 filament assembly rates; bars correspond to the mean and stand deviation for n = 30 molecules for each experiment.

Figure S3. Replicates of high-throughput MMS assays. Scatter plots showing the results of three biological replicates of the high-throughput screen for functional *rad51* variants. The most highly enriched amino acid residue pairs are highlighted.

Figure S4. Replicates of high-throughput sporulation assays. Scatter plots showing the results of three biological replicates of the high-throughput screen for functional *dmc1* variants.

Figure S5. Comparison of interfacial amino acid residues for other members of the Rad51/RecA family. a, Human RAD51 (PDB: 5H1B; (36)). **b,** human DMC1 (PDB: 7C9C; (33)). **c,** *C. elegans* RAD-51 (generated with AlphaFold). **d,** *Drosophila melanogaster* Rad51 (generated with AlphaFold). **e,** *Pyrococus furiosus* RadA (PDB: 1PZN; (85)). **f,** *E. coli* RecA (PDB: 3CMT; (18)). **g,** Interfacial amino acid residue conservation for archaeal RadA. **h,** Naturally occurring RadA interfacial amino acid residue variants.

bioRxiv preprint doi: https://doi.org/10.1101/2024.12.03.626531; this version posted December 4, 2024. The copyright holder for this preprint (which was not certified by peer review) is the author/funder, who has granted bioRxiv a license to display the preprint in perpetuity. It is made available under aCC-BY 4.0 International license.

All Numerical Data underlying the graphs are Provided in a Supplementary Data.xlsx file.

bioRxiv preprint doi: https://doi.org/10.1101/2024.12.03.626531; this version posted December 4, 2024. The copyright holder for this preprint (which was not certified by peer review) is the author/funder, who has granted bioRxiv a license to display the preprint in perpetuity. It is made available under aCC-BY 4.0 International license.

Fig. S1

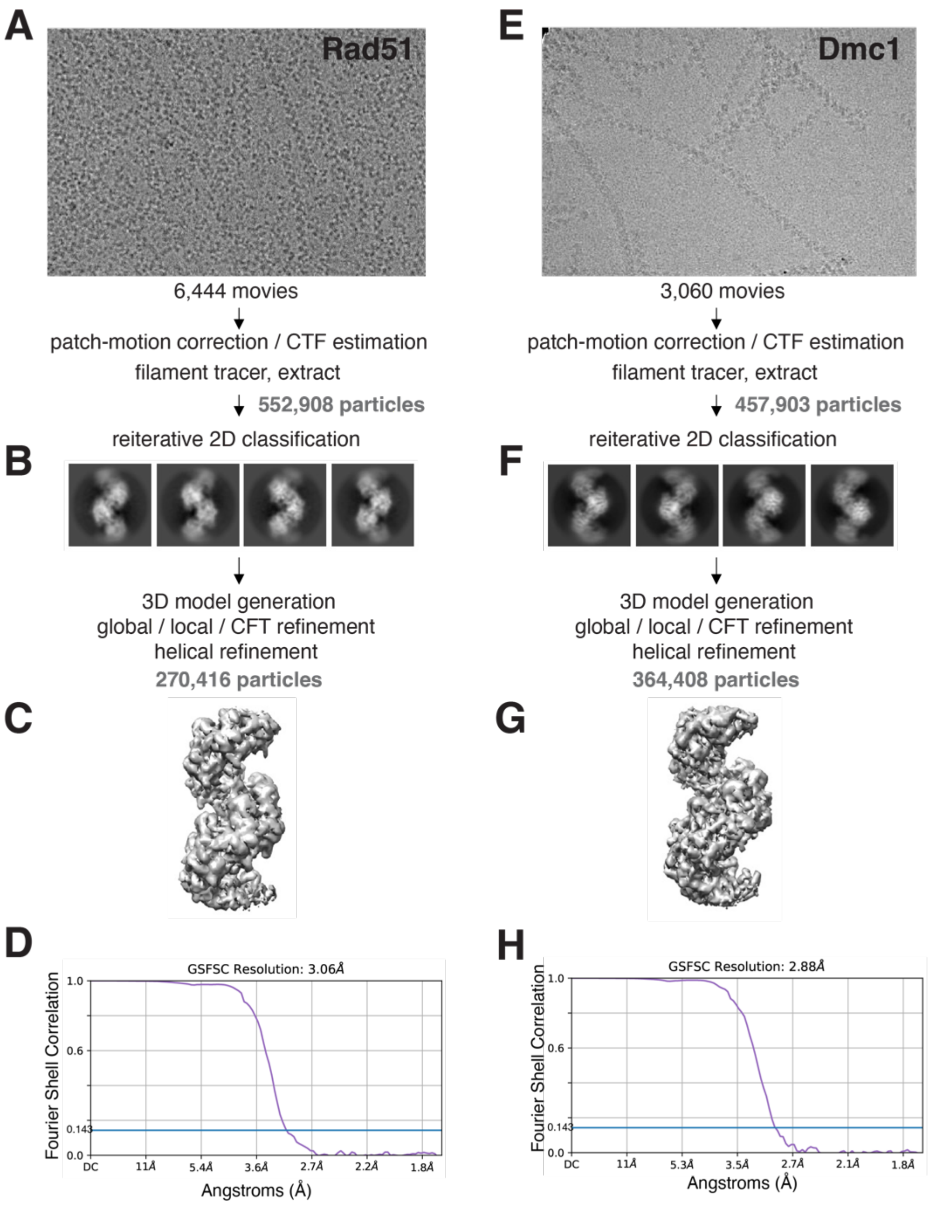

bioRxiv preprint doi: https://doi.org/10.1101/2024.12.03.626531; this version posted December 4, 2024. The copyright holder for this preprint (which was not certified by peer review) is the author/funder, who has granted bioRxiv a license to display the preprint in perpetuity. It is made available under aCC-BY 4.0 International license.

1117 **Fig. S2**

1118

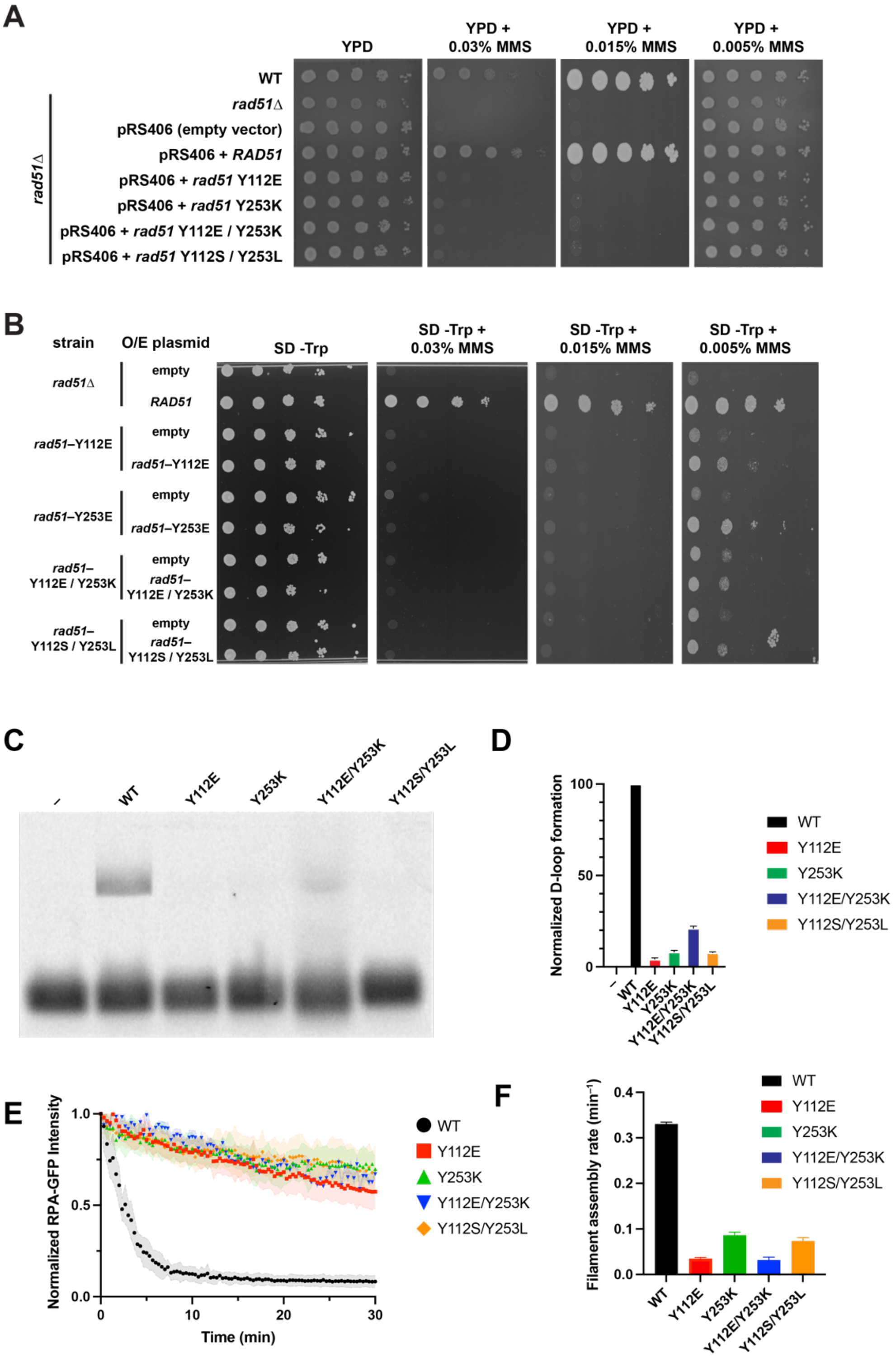

bioRxiv preprint doi: https://doi.org/10.1101/2024.12.03.626531; this version posted December 4, 2024. The copyright holder for this preprint (which was not certified by peer review) is the author/funder, who has granted bioRxiv a license to display the preprint in perpetuity. It is made available under aCC-BY 4.0 International license.

Fig. S3

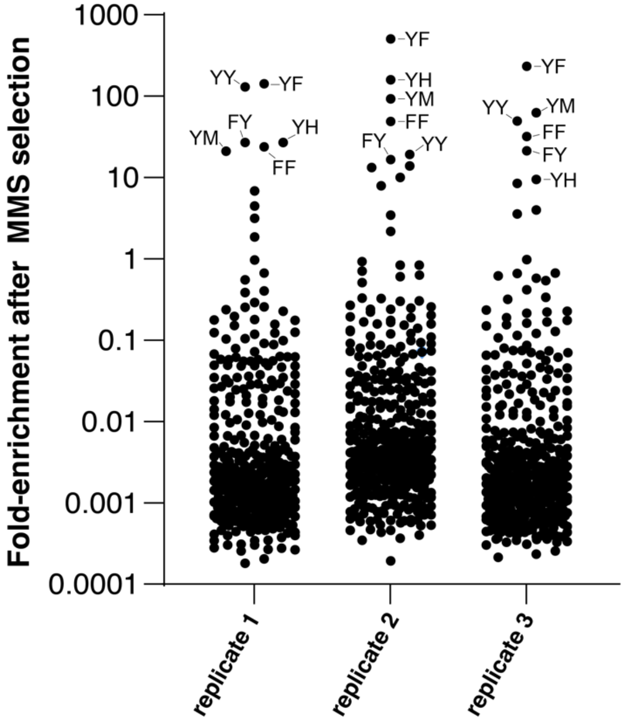

bioRxiv preprint doi: https://doi.org/10.1101/2024.12.03.626531; this version posted December 4, 2024. The copyright holder for this preprint (which was not certified by peer review) is the author/funder, who has granted bioRxiv a license to display the preprint in perpetuity. It is made available under aCC-BY 4.0 International license.

Fig. S4

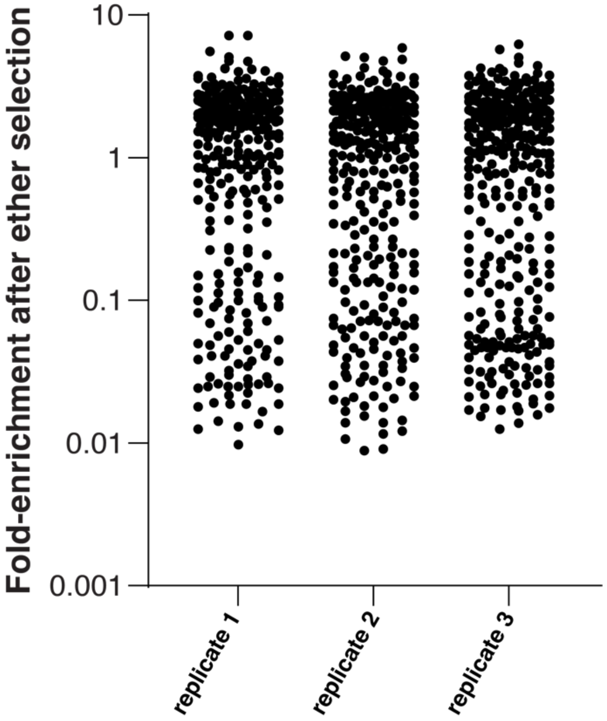

bioRxiv preprint doi: https://doi.org/10.1101/2024.12.03.626531; this version posted December 4, 2024. The copyright holder for this preprint (which was not certified by peer review) is the author/funder, who has granted bioRxiv a license to display the preprint in perpetuity. It is made available under aCC-BY 4.0 International license.

Fig. S5

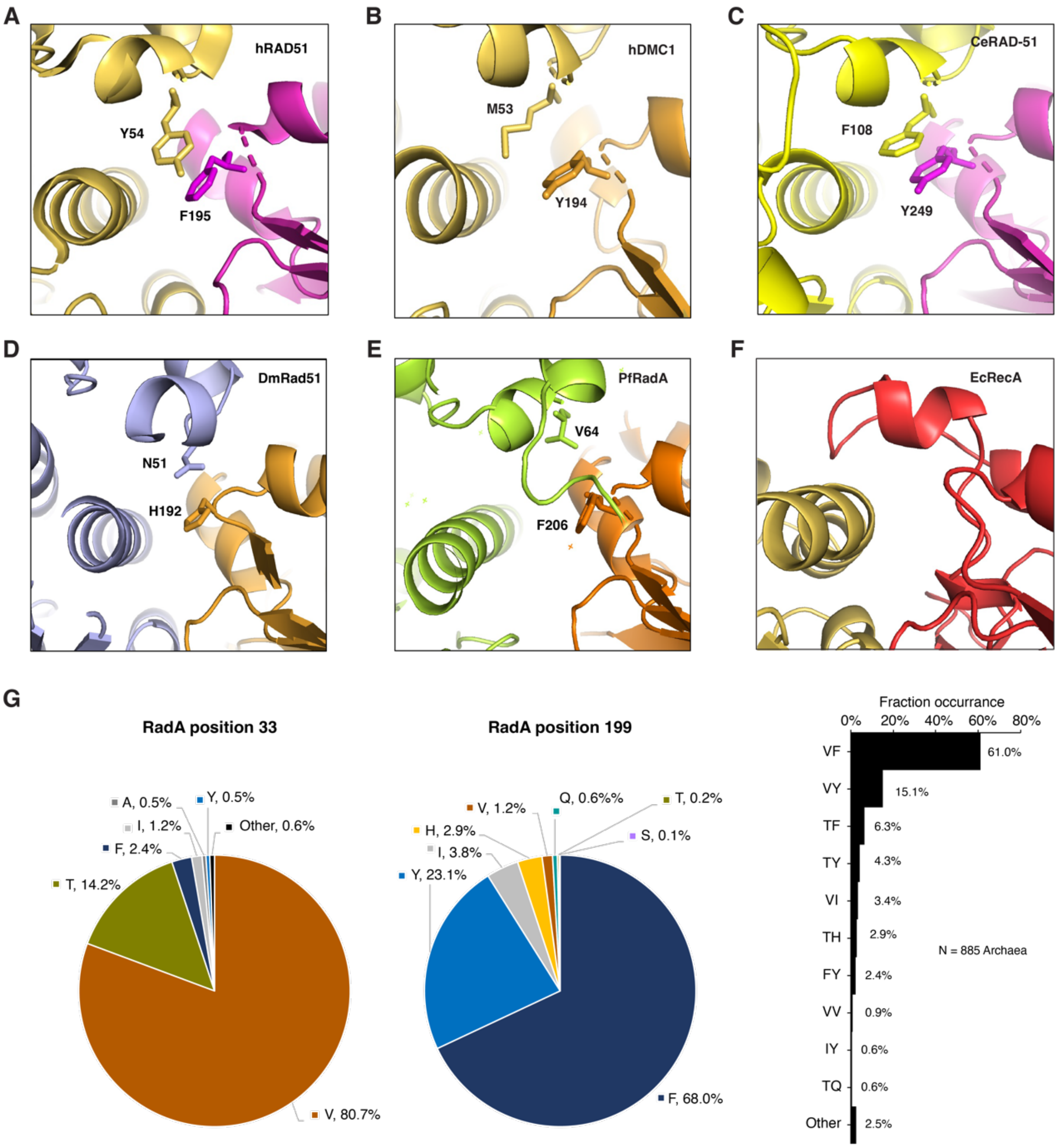

bioRxiv preprint doi: https://doi.org/10.1101/2024.12.03.626531; this version posted December 4, 2024. The copyright holder for this preprint (which was not certified by peer review) is the author/funder, who has granted bioRxiv a license to display the preprint in perpetuity. It is made available under aCC-BY 4.0 International license.

Table S1

	**Rad51**<br>**PDB: 9D46**<br>**(EMD-46550)**	**Dmc1**<br>**PDB: 9D4N**<br>**(EMD-46565)**
**Data collection and processing**		
Microscope	Titan Krios	Titan Krios
Voltage (keV)	300	300
Detector	K3	K3
Magnification	105,000	105,000
Voltage (kV)	300	300
Electron exposure (e^-/Å^2)	51.19	58
Defocus range (μm)	-1 to -2.5	-1 to -2.5
Pixel size (Å)	0.846	0.83
Initial particles picked	552,908	457,903
Final particles used	270,416	364,408
Map resolution (Å)<br>FSC threshold	3.06<br>0.143	2.9<br>0.143
Map resolution range (Å)	3.06-3.7	2.9-3.5
**Refinement**		
Model resolution (Å)<br>FSC threshold	3.1<br>0.143	2.9<br>0.143
Model composition<br>Non-hydrogen atoms	 15,182	 15,018

bioRxiv preprint doi: https://doi.org/10.1101/2024.12.03.626531; this version posted December 4, 2024. The copyright holder for this preprint (which was not certified by peer review) is the author/funder, who has granted bioRxiv a license to display the preprint in perpetuity. It is made available under aCC-BY 4.0 International license.

Protein residues	1924	1866
Ligands	ATP, MG	ATP, MG
B factors (Å^2)		
Protein	89.81	98.53
Ligand	72.12	88.17
R.m.s. deviations		
Bond lengths (Å)	0.004	0.004
Bond angles (°)	0.552	0.517
Validation		
MolProbity score	1.49	1.81
Clash score	9.31	18.18
Rotamer outliers (%)	0	0
Ramachandran plot		
Favored (%)	98.48	97.77
Allowed (%)	1.52	2.23
Outliers (%)	0	0
